# Monitoring Activity of *Spodoptera frugiperda* (Smith) in Different Areas of Maize Crops and Its Pesticide Susceptibility Testing Under Controlled Conditions

**DOI:** 10.1155/jt/6651151

**Published:** 2025-04-20

**Authors:** Alia Tajdar, Chuan Cao, Khalid Abbas, Muhammad Shah Zaib, Hafiz Muhammad Safeer, Syed Muhammad Zaka, Wangpeng Shi, Waqar Jaleel

**Affiliations:** ^1^Department of Entomology, China Agricultural University, Beijing, China; ^2^Department of Entomology, Faculty of Agricultural Sciences and Technology, Bahauddin Zakariya University, Multan, Pakistan; ^3^Entomology Section, Horticultural Research Station, Bahawalpur, Punjab, Pakistan

**Keywords:** cropping season, damage, fall armyworm, insecticide, temperature

## Abstract

Fall armyworm (FAW), *Spodoptera frugiperda* (Smith) (Lepidoptera: Noctuidae), is a polyphagous pest, particularly destructive to maize crops all over the world. It is native to America but has strong flying capabilities, and currently, the FAW has invaded many Asian countries, including Pakistan. Therefore, the current study aims to monitor its activity in four different areas of Pakistan. The damage percentage was recorded in different fields of maize crops caused by FAW. Furthermore, the susceptibility test of four different pesticides was performed against FAW under laboratory conditions. Maximum damage was recorded in the autumn crops that were surrounded by other alternative hosts such as sorghum, potato, jantar, rice chilies, and cotton when compared to the spring crops (with no alternate host in their surroundings). Temperature seems to play an important role in the size of the FAW population and the damage they cause. The results showed that among the four tested insecticides, emamectin benzoate and lufenuron exhibited higher toxic effects, while chlorpyrifos and lambda-cyhalothrin showed lower toxicities against FAW. Additionally, the study revealed an increasing resistance of FAW populations to commonly used insecticides, especially in South Punjab. These findings underscore the urgent need for integrated pest management strategies to address resistance development. Despite the observed resistance, emamectin benzoate remains a viable control option, but proactive resistance management is crucial for its continued effectiveness in long-term FAW control.

## 1. Introduction

*Spodoptera frugiperda* (J.E. Smith) (fall armyworm [FAW]) (Noctuidae, Lepidoptera) is native to South and North America [1]. In 2016, FAW was first reported as a major pest of maize in Nigeria, and it has been reported in more than 28 countries in southern and eastern Africa [2]. Since 2019, FAW has been reported in different Asian countries, such as Pakistan, India, China, and Bangladesh, and has caused severe damage [3–8]. The FAW was recorded to cause 9%–10% damage in the different maize-growing areas of Upper Sindh, Pakistan [5]. The phylogenetic analysis indicated that the rice strain (R-strain) of FAW was feeding on maize crops in Sindh, Pakistan [9]. Furthermore, the existence of FAW on maize from Faisalabad, Punjab, Pakistan, has also been recorded [10]. Montezano et al. (2018) found that FAW larvae can feed on 353 different host plants belonging to 76 plant families, mainly Fabaceae (31), Asteraceae (31), and Poaceae (106). The most favored host of FAW is maize [11]. Moreover, FAW has several generations in a year, significantly depending on temperature [12]. Given regional variations in temperature, farming methods, and natural enemy presence, geographical diversity in FAW management tactics is an important factor to take into account. For example, humidity levels can affect the efficacy of biological control agents such as entomopathogenic fungi, with higher moisture being advantageous for their survival and infection process [13, 14]. Furthermore, the FAW life cycle can be disrupted by using climate-resilient crop varieties and agro-ecological management techniques including crop rotation and intercropping, but their effectiveness depends on regional farming methods and the existence of natural enemies [15, 16]. The need for region-specific monitoring and management techniques is further highlighted by the fact that resistance development and genetic heterogeneity in FAW populations might alter the sensitivity of FAW to insecticides, including emamectin benzoate (EB) [17, 18]. These elements highlight how crucial it is to use adaptable, context-specific, pest management strategies that can change with the environment [19].

The Department of Plant Protection Pakistan (DPP) has formally recognized the existence of the FAW in Pakistan, with the implementation of integrated pest management (IPM) strategies in accordance with FAO guidelines and the declaration of FAW-affected regions under official control. The FAW has been found in districts in Punjab and Sindh provinces that grow maize, with infestation levels as high as 80% in some places [20, 21]. The presence of FAW, *Spodoptera frugiperda*, was confirmed by the DPP and CABI through surveys and research carried out in Okara, Sahiwal, Khanewal, Punjab, and Districts Tando Allah Yar and Mirpur Khas in Sindh [5, 7]. According to the survey, FAW was discovered in the corn-growing belt of the aforementioned districts in Punjab and Sindh, intermingled with other armyworm species such as *Spodoptera litura* and American bollworm, *Helicoverpa armigera* [22]. Regarding the distribution of FAW in other Pakistani provinces, it is known that FAW was initially discovered in Sindh and Faisalabad, which are both in Punjab Province, in 2019-20 [5, 10]. However, the search results that were offered do not include precise data for each province. Other provinces must exercise caution and put monitoring systems in place to identify and control FAW infestations because of the migratory nature of the species and its quick spread.

The control of FAW is mainly dependent on chemical insecticides. It has been reported that chlofenapyr and zeta-cypermethrin have high efficacy for the control of FAW under laboratory conditions in Brazil [23]. Different insecticides: EB, chlorantraniliprole, spinetoram, chlorfenapyr, lambda cyhalothrin, and lufenuron showed high toxicity against FAW under laboratory conditions [24]. The heavy application of insecticides can cause resistance problems [25–27] and harm the environment [28]. In some cases, FAW showed resistance to insecticide classes such as organophosphates (chlorpyrifos), carbamates (carbaryl), benzoylurea (lufenuron), pyrethroids (lambda-cyhalothrin), diamides (chlorantraniliprole), and spinosyn (spinosad) [26, 27, 29, 30].

In this study, the damage of FAW to crop seasons and cropping patterns in different temperature zones areas of south Punjab, Pakistan, during 2020 and 2021. Further, aims are to check the toxicity of commonly used insecticides, chlorpyrifos, lambda-cyhalothrin, lufenuron, and EB and their resistance against FAW.

## 2. Methods

### 2.1. Monitoring FAW

**Figure 1:**
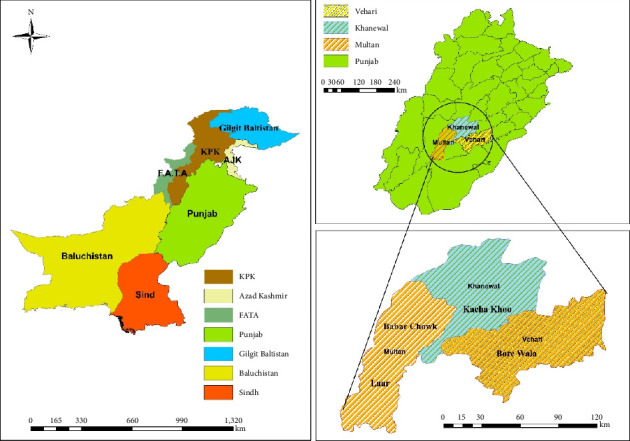
A map of South Punjab in Pakistan illustrating the key sampling sites.

The damage produced by FAW in maize was monitored using the zigzag method and light traps in various fields of south Punjab, Pakistan (2020-2021). Four different sites were visited for monitoring via both methods: Site 1 (Laar) (maize surrounded by chilies, rice, sorghum, jantar, and cotton), Site 2 (Babar Chowk), (maize surrounded by potato, sorghum, citrus, and mango orchard), Site 3 (Kacha khoo) (maize surrounded by cotton, rice, and sorghum), and Site 4 (Bore wala) (Figure 1). For scouting, each field was divided into five blocks with five plants randomly selected and recorded from each block. FAW damage was characterized by ragged whorl leaves, shot holes with ragged leaf infestations, holes in growing cobs and stems, and sawdust-like larval feces [5, 10]. Each field was recorded four times. Moths were taken from light traps on a daily basis between October 8^th^ and October 31^st^, 2020. Larvae collected from corn fields were identified (using their morphological characteristics) and reared in a laboratory as described previously [31]. The following formula was used to calculate the percentage damage of FAW from visited fields [5]:

(1) $$\begin{array}{c} \%\text{Damage}=\frac{\text{damag plants}}{\text{total plants observed}}\times 100. \end{array}$$

### 2.2. Rearing of FAW

The collected specimen of FAW from various visited fields (Table 1) was brought to the laboratory. The larvae were placed individually into Petri dishes, fed with castor bean leaves till pupae, and placed in a jar with moist cotton swab. Sterile cotton soaked in a sugar solution was placed in each jar as a food source for emerged adults. Muslin cloth was hung inside the jar for oviposition of females. The FAW reared till second generations (for the purpose of acclimatization to the castor bean leaves and laboratory condition 25 ± 2°C and 65 ± 5% RH) in the laboratory. After this, eggs were collected, shifted into the Petri dishes, and allowed to hatch. The third instar larvae were used for the insecticide bioassay.

### 2.3. Insecticide bioassay

Four pesticides (see Table 2) were tested against the third instar larvae of FAW. Each pesticide was well-mixed in distilled water using half of the manufacturer's suggested dose, and five to seven concentrations were generated by 50% dilution of the first concentration, and these concentrations were used in the bioassay by serial dilution as described previously [13, 14, 41]. Castor bean leaves were chopped into disks (6 cm) and immersed in various concentrations for 20 s. Leaves were placed on a bench to allow excess moisture to evaporate before being placed individually in a 6-cm petri dish to feed the second instar. Each concentration was tested with 25 larvae and five repetitions. The control group was given with the fresh leaves dipped in distilled water.

Castor bean leaves were chopped into disks (6 cm) and immersed in various treatments for 20 s. Leaves were placed on a bench to allow excess moisture to evaporate before being placed individually in a 6-cm petri dish to feed the second instar. Each treatment was tested with 25 larvae and five repetitions, and each experiment was repeated 6 times [13, 39]. The control group was given with the fresh leaves dipped in distilled water.

### 2.4. Data Analysis

The % damage data by FAW were analyzed using one way ANOVA, and further means were compared via the LSD test at the alpha level 0.05 within single crop duration, whereas the *t*-test was used to compare the damage % between the two seasons. Susceptibility data were analyzed using the EPA Probit analysis program version 1.5 [32] by applying Finney's Probit Analysis [33] to determine lethal concentration (LC_50_) values at 95% confidence limits. The resistance ratios (RRs) was calculated according to the method described elsewhere [34, 35].

## 3. Results

### 3.1. Monitoring damage and Population Size of FAW

**Figure 2:**
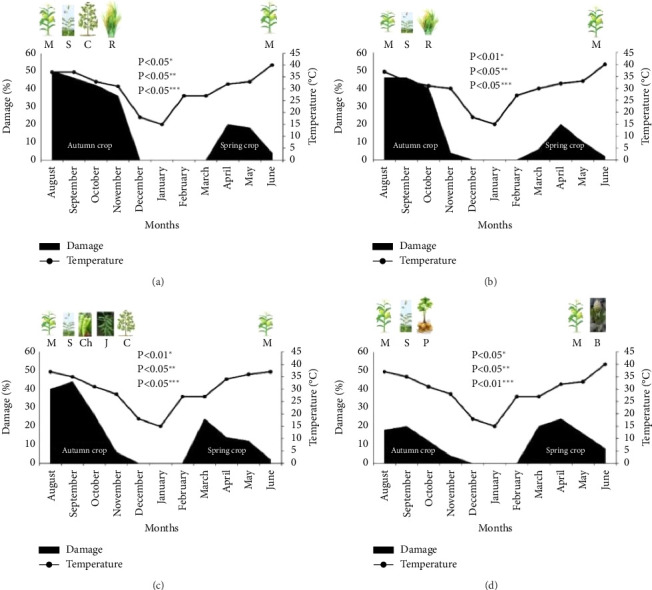
Percentage damage caused by Fall armyworm in relation to temperature and different cropping seasons observed in different locations of South Punjab in 2020 and 2021. (a) Kacha khoo, (b) Bore wala, (c) Laar, and (d) Babar chowk. M: maize, S: sorghum, Ch: chilies, J: jantar, and C: cotton. R: rice, P: potato, and B: barseem. The back-filled area represents the damage percentage of FAW in different months during different cropping seasons, ^∗^FAW damage during different months of the Autumn crop, ^∗∗^FAW damage during different months of the Spring crop, and ^∗∗∗^FAW damage between Autumn and Spring crops.

The damage of FAW was significantly higher in autumn crops when compared to the spring crop at three visited locations: Kacha khoo (autumn vs. spring = 44% vs. 11%, *p* < 0.05, Figure 2(a)), Bore wala (autumn vs. spring = 35% vs. 10, *p* < 0.05, Figure 2(b)), and Laar (autumn vs. spring = 30% vs. 13%, *p* < 0.05, Figure 2(c)). At the fourth location, Babar Chowk, significantly more damage (17%) in spring crop was recorded when compared to that of autumn crop (14%) (*p* < 0.01); however, the difference was small (Figure 2(d)). In general, damage caused by FAW was higher in autumn crops, which may be due to the presence of an alternative host. In addition, the damage analysis during the different months varied and peaked in August and September for the autumn season and April for the spring season (Figure 2). The overall trends of damages were similar to the fluctuation of temperature in all locations, indicating that temperature played an important role in how much damage FAW caused.

**Figure 3:**
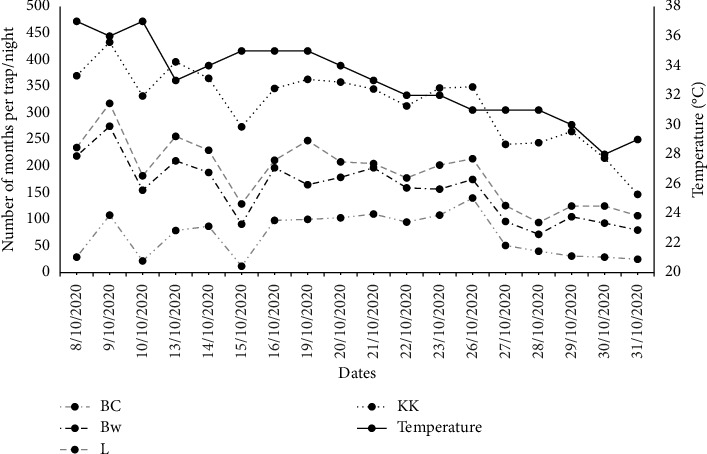
Number of Fall armyworm moths collected by light trap from different areas of South Punjab, Pakistan, in 2020. BC: Babar chowk, BW: Bore wala, L: Laar, and KK: Kacha khoo.

The number of moths varied from area to area, with the highest number of moths collected from Kacha khoo, followed by Laar and Bore wala, while the lowest number of moths were collected from Babar Chowk (Figure 3). This also appeared to be influenced by temperature, as the number of moths per trap/night decreased with a drop in temperature (Figure 3).

### 3.2. Susceptibility

The susceptibility of FAW (*Spodoptera frugiperda*) to various insecticides was assessed in different locations in south Punjab, Pakistan, over the years 2020 and 2021. The insecticides tested included chlorpyrifos, lambda cyhalothrin, lufenorun, and EB. The LC_50_ values and RRs were calculated for each insecticide in each location.

Susceptibility towards chlorpyrifos and EB increased to 85.6% and 60% separately from 2020 to 2021, while susceptibility towards lambda cyhalothrin and lufenuron decreased to 77.2% and 56.4%, respectively. Given the migratory feature of this insect, these changes in susceptibility are more likely due to the genetic background of different migrated populations rather than local usage of insecticides.

In 2020, the RR was 296,130.4345, with Kacha khoo and Bore wala exhibiting RR values of 93,000.000 and 139,869.565, respectively. In 2021, the LC_50_ value for Laar decreased to 4.630 μL/mL, with an RR of 20,130.435, indicating a potential decrease in resistance compared to 2020 (Table 3).

For lambda cyhalothrin, the LC_50_ values in 2020 ranged from 157.300 μL/mL in Laar (RR = 683,913.043) to 284.000 μL/mL in Kacha khoo (RR = 1,234,782.609). Bore wala had an LC_50_ value of 239.400 μL/mL with an RR of 1,040,869.565. In 2021, the LC_50_ value for Laar increased significantly to 1050.000 μL/mL, with an RR of 4,565,217.391, indicating a substantial increase in resistance (Table 3).

The LC_50_ values for lufenorun in 2020 were 7.190 μL/mL in Laar (RR = 31,260.869), 3.360 μL/mL in Kacha khoo (RR = 14,608.696), and 3.670 μL/mL in Bore wala (RR = 15,956.522). In 2021, the LC_50_ value for Laar was 8.420 μL/mL, with an RR of 36,608.696, indicating a slight increase in resistance compared to 2020 (Table 3).

For EB, the LC_50_ values in 2020 were 0.002 μL/mL in Laar (RR = 8.695), 0.000 μL/mL in Kacha khoo (RR = 1.000), and 0.015 μL/mL in Bore wala (RR = 65.217). In 2021, the LC_50_ value for Laar was 0.006 μL/mL, with an RR of 26.087, indicating a slight increase in resistance compared to 2020 (Table 3).

Insecticide susceptibility of FAW populations to different insecticides can be determined using LC_50_ and RR values. In 2021, lambda cyhalothrin RR values were high, suggesting a significant development of resistance among FAW populations. However, the lower LC_50_ values for chlorpyrifos in 2021 indicate a potential decrease in resistance. To mitigate resistance development and ensure sustainable pest management, continued monitoring of insecticide resistance and the development of IPM strategies are imperative (Table 3).

## 4. Discussion

In this study, considering temperature and cropping seasons, we assessed the damage caused by FAW on maize crops in several areas of south Punjab, Pakistan (Figure 1). The results showed that FAW caused more damage in autumn maize crops surrounded by other alternative hosts such as sorghum, potato, jantar, rice, chilies, and cotton, while it caused less damage in spring maize crops because no alternate host was present in the surroundings. Previous research demonstrated that intercropping plays a major role in FAW population dynamics, that is when maize was intercropped with other host crops (barley, beans, cowpea, rice sorghum, wheat, and millet), the damage caused by FAW was larger [36, 37]. However, previous studies also found that the addition of tobacco to garlic and pigeon peas to soybean had detrimental effects on the populations of lepidopteran pests such as *Spodoptera litura, Achaea Janata, Proaerema modicella*, *Myzus persicae*, *Nezara viridula,* and *Heliothis assulta*, as compared to monocropping of garlic and soyabean [38, 39]. This difference might be caused by the presence of chemical effects in intercropping systems as compared to monocropping systems.

Moreover, the data showed that the population size of FAW and the damage caused were temperature dependent, with higher temperatures generally leading to a larger population and more damage. This finding is consistent with previous studies conducted in China, India, Ghana, and Mexico [40–43]. This is possibly due to that increase in temperature will lead to an increase in the development rate of lepidopteran pests such as *Helicoverpa armigera* and *Spodoptera litura* [40–46].

We also tested our field-collected FAW populations against various chemical insecticides and found that our FAW populations were highly susceptible to EB, while they were least susceptible to lambda cyhalothrin under laboratory conditions. EB belongs to the avermectin family, which is an important macrocyclic lactone insecticide that causes DNA damage and apoptosis in the FAW sf-9 cell line [1]. Lambda cyhalothrin belongs to the group pyrethroids, an axonic poison which modulates the voltage-gated sodium channel and delays the closing of this channel [1]. The toxicity of EB was evaluated on different insect species, and it exhibited high toxic effects in killing different lepidopteran species such as *Heliothis virescens*, *Helicoverpa armigera, Spodoptera littoralis*, *Spodoptera exigua, Plutella xylostella, Trichoplusia ni, Pseudoplusia includen*s, *Tuta absoluta*, and *Spodoptera frugiperda* [47–51]. Similarly, in previous studies conducted in China and India, EB showed high toxicity, while lambda cyhalothrin exhibited the least toxic effect against FAW [24, 52–54].

The success of EB as a control strategy is backed by susceptibility data, as highlighted in the study by Patti G Jones et al. [55], which assessed the effectiveness of 114 EB treatment episodes from 2004 to 2008 over 54 farms in the Bay of Fundy, Canada. The study indicated that “Treatment effectiveness varied by geographical region in Pakistan and decreased over time,” showing that while EB was beneficial, its efficacy was not uniform across all regions and over time. While EB has demonstrated efficacy in controlling FAW, the global trend in FAW management is shifting towards more sustainable and environmentally friendly methods as a result of the shift towards IPM strategies. These techniques provide a more thorough approach to pest control while attempting to lessen the dependency on chemical pesticides. EB is known for its selective toxicity, frequently showing less effect on organisms that are not its intended target, such as parasitoids and predators [56]. Because of its high effectiveness against lepidoptera larvae, including the FAW, EB is a significant insecticide. It works by increasing neurotransmitter activity, which causes chloride ions to enter nerve cells and disrupt nerve conduction, which stops larval feeding and causes irreversible paralysis [57].

The fact that EB has a comparatively small effect on nontarget animals, such as parasitoids and predators, is one of its many noteworthy benefits. In an okra habitat, a study assessed the safety of EB 1.9 EC on predatory coccinellids. EB 1.9 EC was found to be safe for coccinellids at all tested concentrations. The largest population was seen at the lowest concentration of 5.00 g a.i. ha^−1^ [58]. According to this, EB can be applied successfully in IPM plans without seriously endangering beneficial predators. Moreover, improvements in EB formulation, such the creation of a pH-responsive metal-organic framework microencapsulation technique, have increased the drug's stability and selectivity. This formulation exhibits higher insecticidal activity, lowers toxicity to nontarget organisms, and lessens the breakdown of EB [59]. This invention is in line with sustainable agriculture's overarching objective, which is to strike a balance between pest management and the maintenance of ecosystem health and biodiversity. These results are in line with other field investigations that showed EB's selective toxicity [60]. For instance, it has been shown that EB is not harmful to beneficial insects such as spiders and lady beetles, which makes it a good option for IPM programs [61]. Natural enemies, which are essential for managing pest populations and lowering the frequency of pesticide applications, can be preserved because of this selective toxicity.

The RRs calculated in this study provide valuable insights into the susceptibility of *S. frugiperda* populations to various insecticides in South Punjab, Pakistan. The data suggest that resistance levels fluctuate significantly depending on the region and year of sampling. These variations might attribute to differences in pest management practices, environmental conditions, and genetic diversity within FAW populations.

In this study, the RR values for chlorpyrifos ranged from 20,130.43 in 2021 to 296,130.43 in Laar in 2020. The field strain of *Spodoptera frugiperda* collected from maize fields in Multan, Southern Punjab, Pakistan, exhibited no resistance to spinosad, showed minimal resistance to EB, and displayed moderate resistance to chlorpyrifos [62]. These findings are in line with previous studies, and toxicity of chlorpyrifos was less than EB against the FAW population from Punjab, Pakistan [63]. Another work reported RR values ranging from 173.91 to 1054.78 in different regions of Brazil [64]. Another study from China reported that RR values of chlorpyrifos were in a range of 1.86–3.16. The significant differences in RRs observed between our study and the findings from Sichuan Province, China, can be attributed to several key factors [65]. High RR values indicate a substantial decline in susceptibility, likely due to extensive and indiscriminate use of this insecticide over the years. The RR values for lambda cyhalothrin were particularly high, with values reaching up to 4,565,217.39 in Laar in 2021. This indicates a significant development of resistance in FAW populations. Previous studies in Punjab have reported increasing resistance to lambda cyhalothrin, with RR values rising from 146.54 to 545.64 folds [62]. The rapid increase in RR values suggests that frequent and widespread use of lambda cyhalothrin in the region may have accelerated the development of resistance [66]. The RR values for lufenuron ranged from 14,608.69 to 36,608.70, indicating a gradual increase in resistance over time. Earlier studies reported a rapid and extremely high level of resistance development in *S. frugiperda* to lufenuron, reaching a 915-fold increase following laboratory selection [67]. This could be due to its relatively infrequent use in the region, reducing the selective pressure for resistance development [68]. For EB, the RR values in this study ranged from 1.00 to 65.22 in 2020, with a slight increase to 26.09 in Laar in 2021. These results are consistent with previous research, which indicates that EB remains effective against FAW, with relatively low resistance levels. Similar results were reported for the EB in field strains of FAW in Egypt [69]. This underscores the importance of IPM approaches, including crop rotation, biological control, and judicious use of insecticides to combat resistance in *S. frugiperda* [70].

## 5. Conclusion

The RRs obtained in this study highlight the increasing resistance of FAW populations to commonly used insecticides in South Punjab, Pakistan. These findings align with previous research, emphasizing the urgent need for IPM strategies to mitigate resistance development. The relatively stable susceptibility to EB suggests that it may still be a viable option for controlling FAW populations, but proactive resistance management is necessary to maintain its effectiveness. Continued monitoring of insecticide resistance, coupled with the adoption of sustainable pest management practices, is essential for the long-term control of FAW and other major agricultural pests.

## Figures and Tables

**Table 1:** Different locations for the monitoring of the fall armyworm population.

Pt. No	Area	Latitude and longitude	Year
1	Kacha khoo	30° 22′ 10.9128″N and 72° 7′ 49.9692″E	2020
2	Bore wala	30°9′27.76″N and 72°40′26.28″E	2020
3	Laar	52°36′55.15″N and 6°44′8.98″E	2020
4	Bore wala	30°9′27.76″N and 72°40′26.28″E	2021

**Table 2:** List of synthetic insecticides, their active ingredients, and mode of action.

Active ingredient	Trade name	Chemical group	Mode of action	Dose (μL/mL)	Manufacturers
Chlorpyrifos	Lorsban 40EC	Organophosphates	Acetylcholinesterase (AChE) inhibitors	500 mL	Arysta life science
Lambda cyhalothrin	Karate 25EC	Pyrethroids	Sodium channel modulators	200 mL	Syngenta (Pakistan limited)
Emamectin benzoate	Proclaim 19EC	Avermectins	Glutamate-gated chloride channel (GluCl) allosteric modulators	100 mL	Syngenta (Pakistan limited)
Lufenorun	Match 50EC	Benzoylureas	Inhibitors of chitin Biosynthesis affecting CHS1	100 mL	Syngenta (Pakistan limited)

**Table 3:** Susceptibility to different insecticides in fall armyworm collected from different areas of south Punjab, Pakistan.

Insecticides	Year	Location	*N*	LC_50_ (μL/mL)^∗^	FL (95%)^≠^	DF^$^	*χ* ^2^	Slope ± SE	RR
Chlorpyrifos	2020	Laar	175	68.110	54.93–84.45	3	1.47	2.63 ± 0.35	296,130.43
	2020	Kacha khoo	150	21.390	15.28–29.94	3	4.46	1.56 ± 0.28	93,000.00
	2020	Bore wala	150	32.170	26.20–39.50	3	1.49	3.09 ± 0.46	139,869.56
	2021		150	4.630	3.45–6.22	1	0.47	3.29 ± 0.92	20,130.43

Lambda cyhalothrin	2020	Laar	150	157.300	91.30–271.20	2	1.08	0.97 ± 0.35	683,913.04
	2020	Kacha khoo	150	284.000	126.80–636.60	1	0.00	1.24 ± 0.78	1,234,782.60
	2020	Bore wala	175	239.400	62.60–915.90	2	0.10	0.21 ± 0.29	1,040,869.56
	2021		150	1050.000	700.00–1577.00	2	3.44	1.94 ± 0.55	4,565,217.39

Lufenorun	2020	Laar	150	7.190	0.80–64.92	1	10.38	3.57 ± 2.38	31,260.86
	2020	Kacha khoo	175	3.360	2.25–5.02	1	0.80	2.44 ± 0.52	14,608.69
	2020	Bore wala	150	3.670	1.83–7.36	3	4.60	1.34 ± 0.36	15,956.52
	2021		175	8.420	5.37–13.20	3	1.12	1.42 ± 0.33	36,608.69

Emamectin benzoate	2020	Laar	150	**0.002**	0.00–0.00	1	0.05	1.29 ± 0.33	8.69
	2020	Kacha khoo	150	0.000^**a**^	0.00–0.00	1	0.02	1.62 ± 0.81	
	2020	Bore wala	175	**0.015**	0.00–0.02	3	3.03	1.28 ± 0.33	65.21
	2021		150	**0.006**	0.00–0.01	3	0.56	0.91 ± 0.33	26.08

*Note:* Kacha khoo's LC_50_ for emamectin benzoate is 0.00023, whereas the FL (95%) of emamectin benzoate is as follows: Laar (0.001–0.004), Kacha khoo (0.00012–0.00047), and Bore wala (0.008–0.029) and (0.003–0.012) for 2020 and 2021, respectively. RR, resistance ratio was calculated for each location as LC_50_ of test generation divided by LC_50_ of susceptible generation in Kacha khoo in 2020. The bold value indicates the fall armyworm (FAW) population that exhibits high susceptibility to emamectin benzoate.

^∗^Lethal concentration.

^≠^Fiducial limit.

^$^Degree of freedom.

^a^ = Standard susceptible population.

## Data Availability

The data that support the findings of this study are available from the corresponding author upon reasonable request.
